# Changes in Eating-Out Frequency according to Sociodemographic Characteristics and Nutrient Intakes among Korean Adults

**Published:** 2020-01

**Authors:** Se-Young JU

**Affiliations:** Major in Food Science, College of Biomedical and Health Science, Konkuk University, Chungju, Korea

**Keywords:** Eating-out frequency, Sociodemographic characteristics, Korean adults, Nutrient intake

## Abstract

**Background:**

The quality of out-of-home foods is an increasingly important issue due to increasing popularity of eating out. The objective of this study was to analyze the relationship of eating-out frequency with general characteristics, dietary habits, and nutrient intakes among Korean adults.

**Methods:**

This study collected data from 2010– 2015 KNHANES. The total number of participants was 33,427 Korean adults aged 19 years and older. All statistical analyses were conducted using SAS software version 9.3.

**Results:**

Eating-out more frequently was associated with younger, unmarried, employed, urban resident, higher income, higher education, and being male. Regarding dietary behavior, subjects with skipping breakfast and taking snack behavior showed a tendency to eat out more frequently. Meanwhile, energy, carbohydrate, protein, fat, and sodium intake were higher in subjects with ≥ 5/week eating-out frequency than those in subjects with < 5/week eating-out frequency.

**Conclusion:**

This study provides important insights into the effect of targeted public health education and policies.

## Introduction

Over the past few decades, eating-out expenditure of household has been increasing due to higher employment of women, greater household income, increase of single family, the lack of time for preparing home-cooked meals, and a decrease in family size. More than 50% of American adults eat out three or more times a week and over 35% eat fast-food meals more than twice a week (1). In UK, more than 27.1% of adults eat meals in full-service or fast-food restaurants once per week or more and 21.1% consume take-away meals at home once per week or more (2). In Korea has also shown an increase trend of eating- out, reporting that over 60% of adults eat out at least once per week (3).

The quality of out-of-home foods is an increasingly important issue due to increasing popularity of eating out. Out-of-home foods are mostly less healthy than home-cook foods because they lead to high energy and fat intakes with low nutritional quality (4). Individuals who frequently eat out of-home meals and fast-food meals have higher BMI and lower serum HDL-cholesterol than those who ate at home (1). In addition, their serum concentrations of plant based nutrients decreased with increasing eating-out (1).

In Kenya (5) and Vietnam (6) out-of-home foods are associated with better diet diversity and quality, particularly Vitamin A and Fe. Larson et al (7) have determined the relationship between dietary intake and choice of restaurant in American young adults and found that more frequent use of full-service restaurants is positively associated with intake of healthy foods (vegetables) compared to that of fast-food restaurants.

In view of increasing out-of-home food consumption, there is a need to examine out-of-home food environment including eating places and types of food consumed outside of the home and sociodemographic factors of out-of- home eaters for supporting healthier diet quality of population. Thus, the objective of this study was to examine the relationship of eating-out frequency with general characteristics, dietary habits, and nutrient intakes among Korean adults based on data from KNHANES (Korea National Health and Nutrition Examination Survey) between 2010~ 2015. The current study can provide important insights into the effect of targeted public health education and policies for encouraging healthier food choices and promoting dietary-related health of people.

## Methods

### Research subjects

**Fig. 1:**
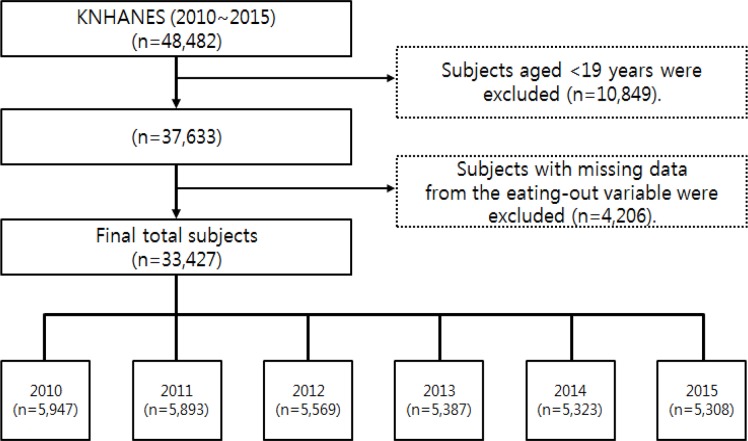
The flow chart of subjects in this study

This study collected data from 2010–2015 KNHANES. KNHANES is a nationwide cross-sectional survey of Korean population containing health behavior interview, health examination, and nutrition survey including a food frequency questionnaire and a 24-hour dietary recall (8,9). The flow chart regarding information of survey subjects is shown in Fig. 1.

This study was approved by the Korea Centers for Disease Control and Prevention Institutional Review Board (IRB approval number: 2010-02CON-21-C, 2011-02CON-06-C, 2012-01EXP-01-2C, 2013-07CON-03-4C, and 2014-12EXP-03-5C). The 2015 KNHANES was exempted from review regarding its research ethics by the Bioethics and Safety Act.

### Eating-out frequency

Eating-out frequency was categorized into six groups from an eating-out variable (L-out-fq): ≥ 1 time/day, 5–6 time(s)/week, 3–4 time(s)/week, 1–2 time(s)/week, 1–3 time(s)/month, and rarely. These were changed to average frequency of eating-out per week using conversion factor shown in Table 1.

**Table 1:** Conversion formula of eating-out frequency among items in KNHANES

***Eating-out Frequency of KNHANES***	***Conversion formula***	***Eating-out frequency per week***
≧2 times a day	{(3+2)/2}*7	17.5
1 time a day	1*7	7
5∼6 times a week	(5+6)/2	5.5
3∼4 times a week	(3+4)/2	3.5
1∼2 times a week	(1+2)/2	1.5
1∼3 times a month	{(1+2)/30}*7	0.47
Seldom (< 1 time a month)	0^*^7	0

### Meal episodes(occasion) and food security status

Meal pattern was classified as breakfast, lunch, and dinner. It was analyzed as skipped or eaten (B+L+D, B+L, B+D, L+D, and others). Snack was grouped Yes or No based on the variable of n_meal. Food security status was assessed using a self-reported questionnaire of dietary life on dietary situation of subjects’ households in the previous year based on KNHANES data (10,11). Food security status was categorized into four groups: 1) food secure group, able to meet essential food and non-food needs without depletion of assets for all family members; 2) mildly insecure group, with minimally adequate food consumption, but unable to afford some essential non-food expenditures without depletion of assets; 3) moderately insecure group, marginally able to meet minimum food needs because of insufficient money; and 4) severely insecure group, often not having enough food to eat because of insufficient money, with large food consumption gaps.

### General characteristics of subjects

General characteristics of subjects were categorized according to gender (male and female), age (19–29 yr, 30–39 yr, 40–49 yr, 50–59 yr, and ≥ 60 yr), residential area (city and rural area), household income (high, middle high, middle low, and low), family number (1–5, and ≥ 6), marital status (married, unmarried), occupation status (employed, unemployed), and educational level (below high school, high school graduate, and college degree or more) based on guidelines of the KNHANES.

### Nutrient intake analysis

To determine the tendency for changes in nutrient intake by eating-out frequency (≥5/week and < 5/week), five nutrient variables (energy, carbohydrate, protein, fat, and sodium) in 24-hour recall data were analyzed by eating-out frequency. Energy contribution of carbohydrate, fat, and protein was analyzed to determine the ratio of each carbohydrate, protein, and fat to energy.

### Statistical analysis

All statistical analyses were performed using complex sampling procedures by applying Strata variables (KSTRATA), clustering variables (PSU: primary sampling unit), and weight variables (Wt_ntr). Analyzed results are presented as weighed percentage (weighted %) or mean and standard error (SE). Chi-square test was used to determine the significance of differences in categorical variables from proc surveyfreq. Significant differences in continuous variables were identified by *p* for trend through proc surveyreg procedure. Multiple correspondence analysis was performed to show relationship between eating-out frequency and general characteristics, meal, and food security variables by weighted percentage. To analyze increase/decrease according to ≥5/week eating-out frequency, eating-out variable was recoded as 1 for “eating-out more than 5 times/week” and 0 for “eating-out less than 5 times/week”. Odd ratios (ORs) and 95% confidence interval (CI) were determined using logistic regression analysis. To analyze nutrient intake for eating-out frequency, LS mean, SE, and *p* for trends were obtained after adjusting for categorical variables such as gender, age, residential area, educational level, household income, household size, occupation status, and marital status. All statistical analyses were conducted using SAS software version 9.3 (SAS Institute Inc., Cary, NC, USA).

## Results

In general characteristics of subjects, gender was nearly balanced (49.4% males vs. 50.6% females). For family number, single family increased from 5.5% in 2010 to 7.8% in 2015. Food secure and moderately food insecure groups significantly increased, whereas mildly food insecure group decreased from 2010 to 2015 (data not shown). Table 2 presents average eating-out frequency per week of subjects according their general characteristics and dietary habit from 2010 to 2015. Males were more likely than females to eat out.

**Table 2:** Average eating-out frequency per week of subjects by the general characteristics and dietary habit according to years

	***2010***		***2011***		***2012***		***2013***		***2014***		***2015***		***Total***		**p *for trend^a^***

	Mean	SE	Mean	SE	Mean	SE	Mean	SE	Mean	SE	Mean	SE	Mean	SE	
Total	4.3	0.1	4.5	0.1	4.1	0.1	4.7	0.1	4.7	0.1	4.8	0.1	4.5	0.04	0.0011(+)
Gender															
Male(n)	5.8	0.2	6.2	0.2	5.5	0.2	6.1	0.2	6.2	0.2	6.2	0.2	6.0	0.1	0.0683(+)
Female(n)	2.8	0.1	2.9	0.1	2.7	0.1	3.2	0.1	3.1	0.1	3.4	0.1	3.0	0.04	<.0001(+)
Age (yr)															
19∼29yr(n)	6.4	0.2	6.8	0.3	5.2	0.2	6.6	0.3	6.1	0.3	6.3	0.2	6.2	0.1	0.2297(−)
30∼39yr(n)	5.2	0.2	5.2	0.2	5.1	0.2	5.8	0.2	5.9	0.2	6.0	0.2	5.5	0.1	0.0005(+)
40∼49yr(n)	4.6	0.2	5.3	0.2	4.9	0.2	5.3	0.2	5.3	0.2	5.7	0.2	5.2	0.1	0.0006(+)
50∼59yr(n)	3.7	0.2	3.9	0.2	3.8	0.2	4.1	0.2	4.5	0.2	4.7	0.2	4.1	0.1	<.0001(+)
More than 60yr(n)	1.4	0.1	1.5	0.1	1.5	0.1	1.9	0.1	1.8	0.1	1.8	0.1	1.7	0.04	<.0001(+)
Education level															
Below high school(n)	1.9	0.1	2.1	0.1	1.9	0.1	2.3	0.1	2.3	0.2	2.1	0.1	2.1	0.1	0.0248(+)
High school graduate(n)	4.2	0.2	4.3	0.2	3.9	0.2	4.4	0.2	4.2	0.2	4.5	0.2	4.2	0.1	0.3140(+)
More than College(n)	5.7	0.2	6.0	0.2	5.3	0.2	5.8	0.2	5.6	0.2	6.0	0.2	5.7	0.1	0.3062(+)
Household income^1)^															
Low(n)	2.5	0.2	2.1	0.2	1.8	0.2	2.3	0.1	2.1	0.2	2.5	0.2	2.2	0.1	0.6888(−)
Middle-low(n)	4.1	0.2	4.3	0.2	3.8	0.2	4.4	0.2	3.9	0.2	4.3	0.2	4.1	0.1	0.7557(+)
Middle-high(n)	4.8	0.2	5.1	0.2	4.7	0.2	5.4	0.2	5.3	0.2	5.0	0.2	5.0	0.1	0.2866(+)
High(n)	5.2	0.2	5.6	0.2	5.0	0.2	5.6	0.2	5.9	0.2	6.1	0.2	5.6	0.1	<.0001(+)
Marital status															
Unmarried(n)	6.7	0.2	7.1	0.3	5.5	0.2	6.7	0.3	6.5	0.3	6.6	0.2	6.5	0.1	0.3400(−)
Married(n)	3.6	0.1	3.8	0.1	3.6	0.1	4.1	0.1	4.2	0.1	4.2	0.1	3.9	0.04	<.0001(+)
Area of residence															
Urban(n)	4.7	0.1	4.8	0.1	4.4	0.1	4.7	0.1	4.8	0.1	5.0	0.1	4.7	0.05	0.1519(+)
Rural area(n)	2.9	0.2	3.2	0.3	2.8	0.2	4.4	0.4	4.0	0.3	3.7	0.3	3.5	0.1	0.0009(+)
Occupation															
Unemployed(n)	2.2	0.1	2.5	0.1	2.3	0.1	2.5	0.1	2.6	0.1	2.7	0.1	2.5	0.05	0.0115(+)
Employed(n)	5.3	0.2	5.4	0.2	5.0	0.1	5.7	0.2	5.6	0.2	5.8	0.2	5.5	0.1	0.0028(+)
Family number															
1(n)	5.1	0.6	3.1	0.2	4.3	0.2	4.9	0.2	4.4	0.3	3.9	0.4	4.5	0.2	0.7526(−)
2(n)	4.7	0.7	3.1	0.2	4.6	0.2	5.2	0.2	4.9	0.3	4.1	0.5	3.5	0.1	0.0001(+)
3(n)	4.2	0.4	2.9	0.2	4.2	0.2	4.8	0.2	4.1	0.3	4.6	0.5	4.6	0.1	0.0147(+)
4(n)	4.0	0.4	3.7	0.2	4.8	0.2	5.3	0.2	5.4	0.3	4.5	0.5	5.1	0.1	0.0286(+)
5(n)	4.5	0.5	3.9	0.2	4.7	0.2	5.4	0.2	4.8	0.3	4.2	0.4	4.7	0.1	0.4373(+)
6(n)	4.6	0.4	3.9	0.2	5.0	0.2	5.4	0.2	4.8	0.3	4.3	0.4	4.2	0.2	0.2301(− +)
Food security^2^)															
Food secure group(n)	4.6	0.2	4.9	0.2	4.3	0.1	4.9	0.2	4.9	0.1	4.9	0.1	4.8	0.1	0.2014(+)
Mildly insecure group(n)	4.1	0.1	4.3	0.2	4.0	0.1	4.6	0.1	4.5	0.1	4.8	0.2	4.4	0.1	0.0001(+)
Moderately insecure group(n)	2.8	0.5	2.9	0.6	2.9	0.4	3.5	0.4	3.7	0.5	3.1	0.3	3.2	0.2	0.7118(+)
Severely insecure group(n)	1.6	0.5	2.4	1.2	3.4	1.3	4.0	0.9	2.2	0.5	2.4	0.6	2.7	0.4	0.3228(+)
Snack															
Yes(n)	4.4	0.1	4.6	0.1	4.1	0.1	4.8	0.1	4.7	0.1	4.8	0.1	4.6	0.05	0.0155(+)
No(n)	2.7	0.3	3.1	0.3	3.5	0.3	3.7	0.3	3.6	0.3	4.1	0.4	3.4	0.1	0.0002(+)
Daily meal pattern^3)^															
B+L+D(n)	4.1	0.2	4.3	0.1	3.9	0.1	4.4	0.1	4.4	0.1	4.5	0.1	4.3	0.1	0.0070(+)
B+L(n)	3.1	0.4	4.2	0.4	3.9	0.4	4.4	0.5	4.3	0.3	4.4	0.4	4.1	0.2	0.0715(+)
B+D(n)	4.1	0.4	3.9	0.4	3.4	0.3	3.9	0.3	3.9	0.3	3.7	0.3	3.8	0.1	0.7120(−)
L+D(n)	5.2	0.2	5.6	0.3	4.8	0.2	5.8	0.2	5.8	0.2	5.8	0.2	5.5	0.1	0.0352(+)
Others(n)	5.4	0.6	4.6	0.4	3.9	0.4	4.4	0.4	5.1	0.4	4.9	0.5	4.7	0.2	0.9364(−)

note:

a *p* for trend by General Lineal Model calculated from Survey procedure of SAS

1) house income was calculated based on total household income of residents and then divided into quartiles from poorest to wealthiest; low, mid-low, mid-high, and high.

2) Food security status was categorized into four groups: 1) food secure group, able to meet essential food and non-food needs without depletion of assets for all family members; 2) mildly insecure group, with minimally adequate food consumption, but unable to afford some essential non-food expenditures without depletion of assets; 3) moderately insecure group, marginally able to meet minimum food needs because of insufficient money; and 4) severely insecure group, often not having enough food to eat because of insufficient money, with large food consumption gaps.

3) meal pattern :B+ L+D(breakfast+ lunch+dinner), B+L(breakfast+ lunch), B+D(breakfast+dinner), L+D(lunch+dinner), others

Total average eating-out frequency per week from 2010 to 2015 generally increased (*p* for trend = 0.0011). For the high household income group, average eating-out frequency per week significantly increased from 5.2 in 2010 to 6.1 times in 2015 (*p* for trend < 0.0001). Odds ratios among general characteristics, dietary habit, and eating-out frequency (≥ 5/week) by years are presented in Table 3.

**Table 3:** Odds ratio among the general characteristics, dietary habit and eating-out frequency (≥5/week) by years

***Variable***	***2010***	***2011***	***2012***	***2013***	***2014***	***2015***	***Total***
Gender (Ref.=Female)							
Male	3.963(3.368–4.663)^a^,^***^	4.368(3.751–5.088)^***^	3.908(3.244–4.709)^***^	4.047(3.494–4.688)^***^	4.033(3.455–4.706)^***^	3.224(2.761–3.765)^***^	3.899(3.660–4.153)^***^
Age (Ref.=19∼29yr)							
30∼39yr	0.564(0.439–0.725)^***^	0.533(0.430–0.661)^***^	0.903(0.683–1.194)	0.879(0.666–1.161)	0.966(0.726–1.286)	0.713(0.563–0.904)^***^	0.739(0.664–0.821)^***^
40∼49yr	0.490(0.380–0.632)^***^	0.515(0.409–0.649)^***^	0.858(0.643–1.143)	0.769(0.590–1.002)	0.916(0.701–1.197)	0.791(0.623–1.004)	0.702(0.632–0.780)^***^
50∼59yr	0.288(0.225–0.368)^*^	0.312(0.243–0.400)^*^	0.436(0.325–0.585)^*^	0.454(0.346–0.596)^*^	0.624(0.462–0.841)^*^	0.461(0.362–0.588)^*^	0.418(0.375–0.466)^**^
More than 60yr	0.061(0.046–0.080)^***^	0.069(0.053–0.090)^***^	0.108(0.079–0.147)^***^	0.117(0.088–0.155)^***^	0.141(0.105–0.189)^***^	0.102(0.079–0.132)^***^	0.097(0.086–0.109)^***^
Education level (Ref.=Below high school)							
High school graduate	3.755(2.920–4.829)^***^	3.677(2.963–4.563)^***^	3.238(2.501–4.191)^***^	3.235(2.670–3.921)^***^	3.005(2.412–3.745)^***^	3.622(2.928–4.481)^***^	3.413(3.115–3.739)^***^
More than College	7.043(5.594–8.867)^**^	6.723(5.470–8.264)^***^	6.823(5.343–8.714)^***^	5.889(4.752–7.299)^***^	5.052(4.037–6.323)^***^	6.486(5.375–7.827)^***^	6.289(5.763–6.863)^***^
Household income (Ref.=Low)							
Middle-low	2.323(1.746–3.091)^*^	3.128(2.349–4.165)^*^	2.711(1.828–4.020)^*^	2.716(2.058–3.586)^***^	3.064(2.200–4.266)^***^	2.921(2.140–3.986)^***^	2.745(2.416–3.119)^*^
Middle-high	3.177(2.324–4.341)^***^	4.572(3.470–6.023)^***^	5.042(3.467–7.332)^***^	4.372(3.266–5.852)^***^	4.959(3.568–6.893)^***^	4.395(3.264–5.919)^***^	4.274(3.762–4.857)^***^
High	3.480(2.558–4.734)^***^	5.442(4.092–7.238)^***^	5.387(3.767–7.703)^***^	4.513(3.353–6.074)^***^	6.619(4.846–9.042)^***^	6.221(4.629–8.359)^***^	5.069(4.467–5.752)^***^
Marital status (Ref.=Married)							
Unmarried	3.984(3.306–4.802)^***^	3.600(3.010–4.305)^***^	2.544(2.014–3.213)^***^	2.565(2.077–3.166)^***^	2.141(1.730–2.649)^***^	2.706(2.213–3.309)^***^	2.830(2.601–3.079)^***^
Area of residence (Ref.=Rural area)							
Urban	2.251(1.651–3.069)^***^	1.950(1.459–2.607)^***^	2.981(2.185–4.065)^***^	1.235(0.883–1.728)	1.360(0.985–1.877)	1.605(1.202–2.144)^***^	2.706(2.213–3.309)^***^
Occupation (Ref.=Unemployed)							
Employed	5.976(4.746–7.525)^***^	5.969(4.877–7.306)^***^	5.339(4.282–6.658)^***^	6.703(5.570–8.066)^***^	7.227(5.889–8.869)^***^	5.526(4.545–6.718)^***^	6.071(5.590–6.594)^***^
Family number(Ref.=1)							
2	0.501(0.344–0.731)^*^	0.664(0.456–0.966)^*^	0.481(0.320–0.724)^*^	0.666(0.470–0.944)^*^	0.856(0.612–1.199)	0.707(0.499–1.003)	0.644(0.553–0.750)^*^
3	0.629(0.462–0.855)^*^	0.912(0.590–1.409)	0.632(0.403–0.992)^*^	0.804(0.536–1.208)	0.897(0.628–1.281)	0.765(0.535–1.096)	0.763(0.650–0.897)^*^
4	0.729(0.517–1.028)	0.946(0.619–1.446)	0.843(0.532–1.337)	0.924(0.614–1.391)	0.976(0.660–1.442)	0.835(0.588–1.186)	0.866(0.735–1.021)
5	0.682(0.467–0.998)^*^	1.151(0.725–1.827)	0.694(0.408–1.181)	0.918(0.563–1.495)	0.847(0.530–1.355)	0.617(0.410–0.927)^*^	0.799(0.662–0.966)^*^
≥6	0.577(0.336–0.991)^*^	0.760(0.391–1.478)	0.749(0.404–1.391)	0.839(0.477–1.478)	0.717(0.433–1.188)	0.864(0.499–1.496)	0.734(0.577–0.935)^*^
Food insecurity (Ref.=Food secure group)							
Mildly food insecure group	0.871(0.743–1.022)	0.794(0.674–0.936)^***^	0.801(0.669–0.959)^*^	0.840(0.724–0.974)^*^	0.995(0.862–1.149)	1.005(0.860–1.174)	0.877(0.822–0.935)^***^
Moderately insecure group	0.518(0.284–0.944)^*^	0.357(0.203–0.626)^***^	0.424(0.248–0.724)^*^	0.525(0.365–0.754)^*^	0.523(0.346–0.791)^*^	0.440(0.292–0.664)^**^	0.462(0.382–0.560)^***^
Severely insecure group	0.323(0.081–1.284)	0.106(0.013–0.851)^***^	0.379(0.113–1.274)	0.394(0.158–0.984)^*^	0.523(0.346–0.791)^***^	0.569(0.286–1.135)	0.387(0.251–0.596)^**^
Snack (Ref.=No)							
Yes	2.396(1.775–3.234)^***^	2.066(1.586–2.691)^***^	1.519(1.177–1.960)^**^	1.519(1.177–1.960)^**^	1.580(1.125–2.219)^**^	1.168(0.853–1.600)	1.714(1.518–1.936)^***^
Daily meal pattern (Ref.=B+L+D)							
B+L	0.693(0.454–1.056)	0.897(0.613–1.311)	0.954(0.653–1.395)	0.848(0.590–1.221)	0.990(0.705–1.390)	0.864(0.617–1.209)	0.876(0.754–1.016)
B+D	0.933(0.676–1.287)	0.732(0.483–1.110)	0.886(0.617–1.273)	0.734(0.545–0.989)^**^	0.702(0.498–0.991)^***^	0.710(0.504–1.001)	0.775(0.671–0.895)^***^
L+D	1.839(1.520–2.223)^***^	1.907(1.530–2.376)^*^	1.526(1.237–1.882)^***^	1.740(1.485–2.039)^***^	1.657(1.386–1.982)^***^	1.739(1.435–2.108)^***^	1.733(1.604–1.872)^**^+
Others	1.765(1.129–2.759)^*^	1.678(1.066–2.643)^***^	0.968(0.560–1.671)	1.213(0.777–1.894)	1.325(0.854–2.056)	1.201(0.823–1.751)	1.318(1.092–1.590)^***^

note:

a Odds ratio(95% CI: Confidence Interval),

* *P*<0.05,

** *P*<0.01,

*** *P*<0.001

**Fig. 2:**
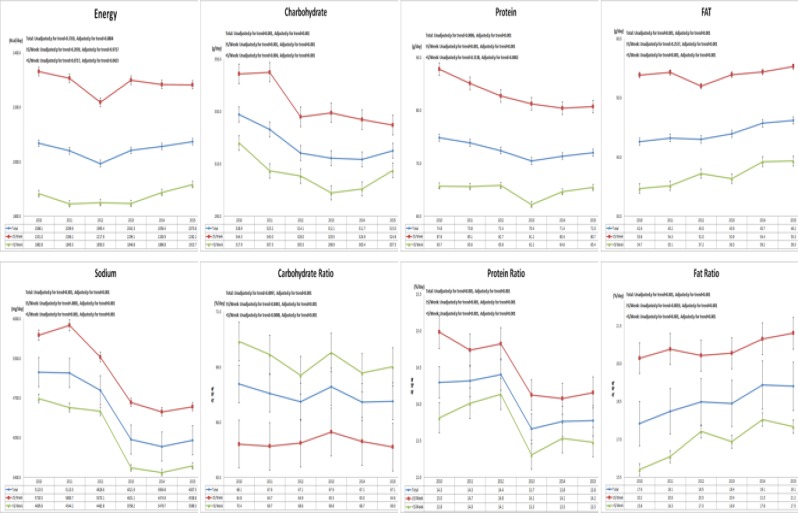
Comparison of the energy, macronutrient, sodium intakes and energy contribution from eating-out frequency by 2010~2015 ^1)^ Mean and S.E were adjusted by gender, age, residential area, educational level, income level, job and marital status. ^2)^ *P* for trend obtained by command of proc regress from SUDAAN.

For gender, ≥ 5/week eating-out frequency in males increased about 3.2~4.4 times more than that in females by years. Subjects with higher level of education, higher household income, and urban residence ate out more than 5 times/week. Regarding marital status, eating-out frequency of unmarried subjects was 2~4 times higher than that of married subjects. In addition, employed subjects showed 5.3~7.2 times higher eating-out frequency than unemployed subjects. Lastly, subjects with skipping breakfast (L+D) behavior ate out 1.5~2 times higher than those with taking all three meals (B+L+D). Figure 2 presents energy, carbohydrate, protein, fat, and sodium intakes and energy contribution from eating-out frequency. Energy intake was not significantly changed from 2010 to 2015. Intake of carbohydrate, protein, and sodium generally decreased by years (unadjusted *p* for trend < 0.001), whereas intake of fat significantly increased by years (un-adjusted *p* for trend < 0.001). Energy contribution ratio of carbohydrate and protein significantly decreased (adjusted *p* for trend < 0.001), while that of fat significantly increased by years (adjusted *p* for trend < 0.001). Regarding eating-out frequency, the intake of energy, carbohydrate, protein, fat, and sodium of subjects with eating-out frequency of ≥ 5/week were higher than those with eating-out frequency of < 5/week.

## Discussion

This study analyzed the relationship between eating-out frequency and general characteristics, dietary habits, and nutrient intakes from 2010 to 2015. We found that eating-out more frequently was associated with younger, unmarried, employed, urban resident, higher income, higher education, and being male. For dietary behavior, subjects with skipping breakfast and taking snack behavior showed a tendency to eat out more frequently. Meanwhile, energy, carbohydrate, protein, fat, and sodium intakes of subjects with eating-out frequency of ≥ 5/week were higher than those with eating-out frequency < 5/week.

There have been several studies in developed countries focusing on trends of eating-out related to this study. In cross-sectional dietary data of eating-out trend 1987~2000 in America, subjects who eat out more frequently are generally males, younger aged, and employed (12). This finding was consistent with results of another eating pattern study which reported that frequent eating-out was positively related with being male, younger, non-white, educated, and employed (13). A large study from 10 European countries also presented that variables of sedentary lifestyle, higher education, young age, and male were positively associated with more frequent eating-out (14). This finding is consistent with a recent study on eating-out patterns in four Nordic countries that reported individuals who were young, urban resident, higher educated, and had greater income generally ate out more frequently (15). Mills et al (16) have also reported the relationship between sociodemographic characteristics and eating-out frequency in population of United Kingdom. They found that eating-out more frequently was positively associated with being male, working overtime, higher income and education, whereas consuming more frequent home-cooked meals was associated with being female, not working overtime, older. Interestingly, higher socioeconomic status based on household income and educational level was positively related to more frequent consumption of home-cooked meals and lower frequency of eating takeaways as well as more eating-out frequency. This means that the place and variety of eating-out are closely associated with diet quality of out-of-home foods.

In general, a higher income is associated with more disposable income which may lead to a higher frequency of eating-out (4, 17). Moreover, increasing household income from dual-career families is also associated with the lack of time for preparing home-cooked meals and busy lifestyle, resulting in more frequent eating-out or use of convenience foods.

Several studies have reported that the relationship between household size and fast-food consumption (18–21). They found that smaller household size tended to show more fast-food consumption. This finding would be related to increase single families as well as time constraints and convenient life styles.

We found that subjects with skipping breakfast behavior tended to eat out more frequently than others. Eating-out more is closely associated with individuals with more frequent skipping breakfast behavior based on data from 2007~2009 KNHNES (22). The result from trend of eating-out occasion of Korean adults in 1998~2012 has shown that the proportion of frequency of eating at home is significantly decreased by years (23). This was associated with skipping breakfast and eating breakfast away from home because eating breakfast and dinner mostly take place at home compared to eating lunch. This indicates that lack of time may be related to not enough time to prepare home-cooked breakfast. Frequently eating-out is positively related to being male and working overtime (16). Breakfast is often considered the most important meal of the day because it provides energy for activities of the day. Skipping breakfast may be a cause of obesity due to excessive food energy intake from other meals. Nonbreakfast eating occasions on no-breakfast day contained higher energy than those on breakfast day from NHANES 2005~2010 data (24).

Those with eating-out frequency of ≥5/week had higher energy, carbohydrate, protein, fat, and sodium intakes and energy contribution ratio of protein and fat than those with eating-out frequency of < 5/week. Especially, fat intake and the energy contribution ratio of fat were gradually increased while other nutrients stayed similar levels or decreased by years. Todd (25) has examined the changes of food away from home, total energy intake, and intakes of fat, saturated fat, cholesterol and fiber among working-age adults in the US from 2005 to 2014. She found that more eating-out was associated with higher in-takes of fat and saturated fat but lower the intake of fiber. Out of home eating is associated with greater daily energy intake in Europe (14). Intakes of energy, protein, fat, and carbohydrates are significantly higher at home than those away from home, while energy contribution of fat is the greatest outside home (26).

## Limitations

First, results of this study may not reflect usual dietary intake of Korean population because data used a single day of 24-hour recall intake. However, it is still a recognized method to assess dietary intake of population as a large scale national survey. Second, the diversity of survey questions used to analyze trends of eating-out has not been explored. Despite these limitations, a major strength of this study was a nationally representative sample of 33,427 Korean adults was analyzed for eating-out frequency and general characteristics, dietary habits to understand the relationship between eating-out frequency and sociodemographic factors using data of recent years.

## Conclusion

This study analyzed the relationship between eating-out frequency and sociodemographic factors and nutrient intakes from 2010 to 2015. Eating-out more frequently was associated with younger, unmarried, employed, urban resident, higher income, higher education, and being male. These findings may be used to as basic information to promote dietary-related health and make healthier food choices, particularly focusing on individuals who consume out-of-home foods frequently, such as those who are younger, unmarried, employed, urban resident, higher income, higher education, and being male.

## Ethical considerations

Ethical issues (Including plagiarism, informed consent, misconduct, data fabrication and/or falsification, double publication and/or submission, redundancy, etc.) have been completely observed by the authors.
